# Infusion of 2.5 meq/min of lactic acid minimally increases CO_2_ production compared to an isocaloric glucose infusion in healthy anesthetized, mechanically ventilated pigs

**DOI:** 10.1186/cc13098

**Published:** 2013-11-11

**Authors:** Alberto Zanella, Marco Giani, Sara Redaelli, Paolo Mangili, Vittorio Scaravilli, Valentina Ormas, Marco Costanzi, Mariangela Albertini, Giacomo Bellani, Nicolò Patroniti, Antonio Pesenti

**Affiliations:** 1 Department of Medical Sciences, University of Milano-Bicocca, Ospedale San Gerardo Nuovo dei Tintori, via Donizetti 106, 20900 Milan, Monza, Italy; 2 Dipartimento di Scienze Veterinarie e Sanità Pubblica, Università degli Studi di Milano, Via Celoria, 10, 20133, Milan, Italy

## Abstract

**Introduction:**

Blood acidification by lactic acid infusion converts bicarbonate to CO_2_. This effect can be exploited to increase the transmembrane PCO_2_ gradient of an extracorporeal membrane lung, resulting in a significant increase of extracorporeal CO_2_ removal. Lactic acid, however, is an energetic substrate and its metabolism might increase total body CO_2_ production (VCO_2_), limiting the potential beneficial effects of this technique. The aim of our study was to compare VCO₂ during isocaloric infusion of lactic acid or glucose.

**Methods:**

Six pigs (45 ± 5 kg) were sedated and mechanically ventilated. Estimated caloric needs were 2,300–2,400 Kcal/die (95 to 100 Kcal/h). A sequence of two steps lasting four hours each was performed: 1) Glucose, 97 kcal/h were administered as 50% glucose solution, and 2) Lactic Acid, approximately 48.5 kcal/h were administered as lactic acid and approximately 48.5 kcal/h as 50% glucose solution. This sequence was repeated three times with two-hour intervals. Every hour VCO₂, arterial blood gases and lactate were measured. Blood glucose level was kept constant by titrating an insulin infusion, ventilation was adjusted to maintain arterial PCO_2_ at 50 mmHg, a normal value for our animal model.

**Results:**

During Lactic Acid steps VCO_2_ increased less than 5% compared to the Glucose steps (282 vs. 269 ml/min, *P* <0.05); blood glucose did not differ between the two groups (respectively 101 ± 12 vs. 103 ± 8 mg/dl). Arterial lactate was always lower than 3 mmol/L. Arterial pH was lower during Lactic Acid steps (7.422 vs. 7.445, *P* <0.05).

**Conclusions:**

Replacing 50% of the caloric input with lactic acid increased total CO_2_ production by less than 5% compared to an equal caloric load provided entirely by a 50% glucose solution.

## Introduction

Partial extracorporeal CO_2_ removal (ECCO_2_R), initially introduced in the late 1970s [1-5], allows ultra-protective mechanical ventilation, and might help limit Ventilation-Induced Lung Injury (VILI) in Acute Respiratory Distress Syndrome (ARDS) patients [6]. Moreover, it may enable awake hypercapnic patients [7,8] or patients awaiting lung transplantation to avoid endotracheal intubation [7,9]. Despite major improvements in extracorporeal technology, moderate/high extracorporeal blood flow rates (500 to 1,000 ml/minute) [10,11] are still required to remove a significant fraction (for example, 50%) of the total CO_2_ production of an adult patient. This implies the use of large diameter vascular catheters and specific technical requirements that limit the application of the procedure. The rate of CO_2_ removal is limited by the fact that most of the CO_2_ in blood (approximately, 90%) is present as bicarbonate ion that cannot cross the artificial lung membrane, which is permeable to gases but not to solutes and water. Acid infusion shifts bicarbonate dissociation to the gaseous CO_2_ form, dramatically increasing the transmembrane pressure gradient and thus increasing extracorporeal CO_2_ removal. Regional extracorporeal infusion of lactic acid before the artificial lung has proved to effectively increase the efficiency of an extracorporeal CO₂ removal system in an experimental setting [12,13]. This technique may allow the removal of more than 100 ml/minute of CO_2_ from an extracorporeal blood flow as low as 250 ml/minute [14]. Such a low blood flow may be achieved with vascular accesses and technology similar to the ones used for continuous renal replacement therapy in the intensive care unit. Lactic acid, however, is an energetic substrate and its metabolism, producing CO₂, may potentially reduce the clinical benefit of extracorporeal CO₂ removal [15-17]. The present study was designed to assess, in a swine model, the impact of lactic acid infusion on whole body CO₂ production compared to an isocaloric glucose infusion.

## Materials and methods

### Experimental setting

Animal care and treatment were conducted in accordance with the Institutional Guidelines for the Care and Use of Laboratory Animals (University of Milan) and in compliance with national laws and policies (D.L. n.116 G.U., suppl. 40, 18/02/1992; Circolare n.8, G.U., 14/07/1994), under the supervision of the veterinarian responsible for laboratory animal welfare. The protocol was approved by the University of Milan and MIUR (Ministero dell’Istruzione, dell’Università e della Ricerca).

Six female Large White pigs (weight 44.9 ± 5 kg) were initially sedated with an intramuscular injection of medetomidine (Domitor® PFIZER ITALIA Srl (DIV.VET.), Latina, Italy, 0.03 mg/kg) and tiletamine-zolazepam (Zoletil® VIRBAC Srl, Milan, Italy, 4 mg/kg); after catheterization of an ear vein, anesthesia was induced with propofol (Propofol Kabi® Fresenius Kabi Italia S.r.l. Isola della Scala, Verona, 2 to 2.5 mg/kg) to allow endotracheal intubation and mechanical ventilation (Servo 900C, Siemens-Elema AB, Sweden). Anesthesia was then maintained with continuous intravascular infusion of sodium thiopental (Pentothal Sodium® HOSPIRA SpA, Liscate (MI), Italy, 10 to 25 mg/kg/h), fentanyl (Fentanest® Pfizer Italia S.r.l., Latina, Italia, 50 to 200 mcg/h) and rocuronium (Esmeron® MSD Italia S.r.l., Roma, Italia, 30 to 100 mg/h). A Foley urinary catheter (8 to 10 Fr) was inserted and connected to a urine collection bag. Before surgery antibiotic prophylaxis with ceftriaxone (Fidato® Fidia Farmaceutici S.p.A., Abano Terme (PD), Italia, 2 g) and gentamycin (Gentamicina Pensa® Pensa Pharma S.p.A., Milano, Italia, 80 mg) was administered. The right femoral artery and left internal jugular vein were surgically cannulated for pressure monitoring, blood gas analysis and drug infusion. Following administration of an unfractioned heparin bolus (Epsoclar® Mayne Pharma Srl, Napoli, Italia, 200 U/kg), pigs were connected to a veno-venous extracorporeal circuit after surgical cannulation of the right external jugular vein with a 14-Fr catheter (Medtronic, Minneapolis, MN, USA) for blood drainage and of the right femoral vein with a 10 to 12-Fr cannula for reinfusion. The extracorporeal circuit, required to infuse a concentrated (40%) lactic acid solution, consisted of a centrifugal pump (Jostra rotaflow, Maquet, Hechingen, Germany), a gas trap, a polysulphone membrane dialyzer (F8HPS, Fresenius, Bad-Homburg, Germany) and ^1^/_4_ to ^3^/_16_ inch inner-diameter tubing. No membrane lung was placed in the extracorporeal circuit. The outlet port of the dialyzer was connected to the inlet port to create a closed system with the dialysate flowing countercurrent to blood flow.

**Figure 1:**
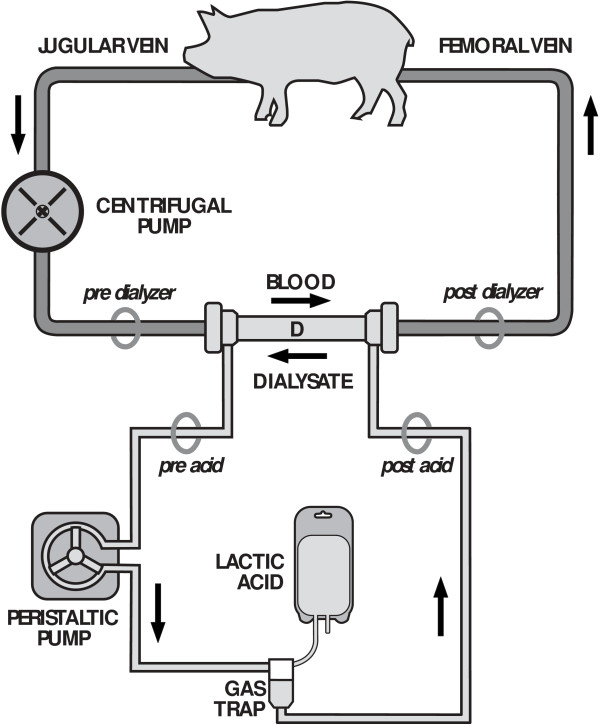
**Outline of the experimental setup.** DF, dialyzer hemofilter. Circles represent the withdrawal sites.

The experimental setup is outlined in Figure 1. The extracorporeal blood flow was set to 250 ml/minute and the dialysate flow at 300 ml/minute. A target activated clotting time (Hemocron Jr. Signature, ITC, Edison, NJ, USA) value of 250 to 300 sec was maintained by titrating a continuous infusion of unfractioned heparin.

During the experiment, the tidal volume was adjusted in order to maintain an arterial PCO_2_ of 50 mmHg, which is a physiologic value for both our animal model and the chronic hypercapnic patients and corresponds to a nearly neutral pH due to the high bicarbonate content of their blood. The inspiratory oxygen fraction was 40%; body temperature was controlled at 38°C through external physical methods. An intravenous infusion of a rapid-acting insulin analog was titrated to keep blood glucose levels constant (target = 100 mg/dl). At the end of the experiment all the pigs were sacrificed with a bolus injection of KCl (40 mEq) in the central venous line.

### Study design

The experiment consisted of two steps, lasting four hours each, which differed only for the caloric source provided:

– Glucose step: 97 kcal/h were administered, through the central venous catheter, as a 50% glucose solution (52 ml/h)

– Lactic Acid step: 48.5 kcal/h were administered (34 ml/h) as a L-(+)-Lactic acid solution approximately 40% in H_2_O (Sigma-Aldrich Corp. St. Louis, MO, USA) into the gas trap of the recirculating dialysate [14], 48.5 kcal/h as 50% glucose solution (26 ml/h) were infused through the central venous catheter), for a total caloric load of 97 Kcal/h. The infused lactic acid amounted, therefore, to 2.5 mEq/minute.

**Figure 2:**
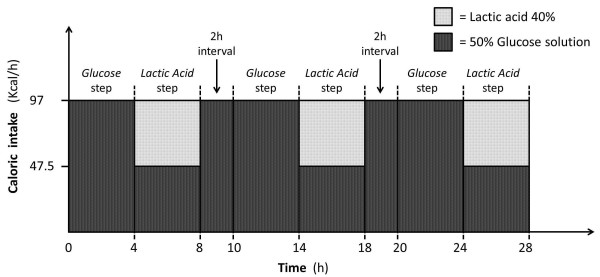
Schematic representation of the experiment timeline.

These two steps, always performed in a preset sequence (Glucose followed by Lactic Acid) were repeated three times with a two-hour interval following the Lactic Acid step to allow a complete clearance of the infused lactate (Figure 2).

The total caloric intake during the whole study period was approximately 2,330 kcal/day, consistent with the estimated basal metabolic rate of anesthetized and curarized swine weighing approximately 45 kg [18].

Every hour (except during the two-hour interval after discontinuation of lactic acid infusion) we recorded: total CO_2_ production (VCO_2_), body temperature, arterial blood gases, blood glucose and arterial lactate level, hemodynamic parameters (heart rate, systemic arterial pressure, central venous pressure) and ventilatory parameters (tidal volume, respiratory rate, minute ventilation, airway pressures). All the blood gas analyses were performed with the ABL 800 FLEX (Radiometer, A. De Mori, Milano, Italy) which is equipped for the automatic determination of pH, blood gases, oxymetry, electrolytes, glucose and lactate. Fully automated quality controls are carried out; every four hours a manual calibration was performed. The ABL 800 measures the lactate concentration in the plasma. We calculated the ventilation of the dead space (VD) according to the standard equation: VD = VE * (PaCO_2_-PeCO_2_/PaCO_2_). PeCO_2_ = partial pressure of expired mixed CO_2_. Alveolar ventilation (VA) was calculated as follows: VA = VE - VD.

Total CO_2_ production (VCO_2_) was calculated as the product of expired CO_2_ concentration and minute ventilation (VE). The expired CO_2_ concentration was measured through an infrared CO_2_ analyzer (WMA-4, GMR Strumenti SAS, Florence, Italy) sampling at 400 ml/minute from a 20-liter mixing box connected to the exhaled gas exhaust port of the ventilator. VE was read from the ventilator, which was calibrated against a pneumotachograph (MLT300L, ADInstruments, Sydney, QLD, Australia), whose accuracy was regularly checked with a calibrated syringe.

### Statistical analysis

Statistical analysis was performed using the SigmaPlot 11.2 statistical software (Systat Software Inc., Chicago, IL, USA). Data are expressed as means ± standard deviations. To compare data of Glucose steps versus Lactic Acid steps a paired Student *t*-test was used. A paired-t analysis was performed to increase the statistical power, eliminating the confounding factor of the between-pig variation; actually a much larger sample would have been required to reject the null hypothesis without a paired analysis. The Shapiro-Wilk test was used to assess normality of the samples. A two-way ANOVA was performed to assess whether the *time* factor had an impact on VCO_2_ during the different steps.

## Results

A total of 16 two-step sequences (Glucose - Lactic Acid), were conducted on six animals. No complications related to lactic acid infusion were reported. In two pigs the study was interrupted before the third two-step sequence, in one case due to abrupt clotting of an extracorporeal cannula and in the other case due to severe hemodynamic instability.

**Figure 3:**
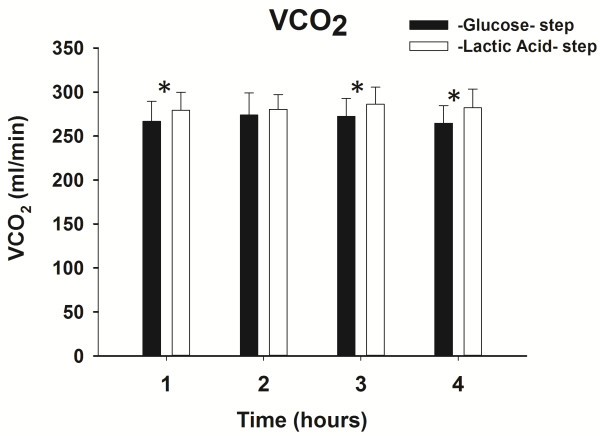
**VCO**_**2 **_**during Glucose and Lactic Acid steps over time.** *: *P* <0.05 versus the glucose step at the same time.

The average total CO_2_ production was 269 ± 22 and 282 ± 19 ml/minute, respectively, during Glucose and Lactic Acid infusion (*P* <0.05). The increase in VCO_2_ during the Lactic Acid step was 4.8%. This difference in VCO_2,_ though quantitatively small, was, however, statistically significant at the first, third and fourth hours of each step (see Figure 3), but not at the 2^nd^. The time factor did not affect VCO_2_ (*P* = 1.00), which remained stable over time during both the Glucose and Lactic Acid steps.

Arterial lactate was higher (2.6 ± 0.6 vs. 0.4 ± 0.2 mmol/l, *P* <0.05), arterial pH was lower (7.422 ± 0.03 vs. 7.445 ± 0.02, *P* <0.05) and HCO_3_^-^ was lower (32.2 ± 2.3 vs. 33.9 ± 1.7 mmol/L, *P* <0.05) during the Lactic Acid steps. Arterial PCO_2_ (50.1 ± 1.4 vs. 50.3 ± 1.5 mmHg, *P* = 0.54) and arterial glucose (107 ± 15 and 104 ± 13 mg/dl, *P* = 0.25) were kept constant; as was the amount of insulin needed to maintain target blood glucose (1.2 ± 0.8 and 1.3 ± 1 U/h during Lactic Acid and Glucose, respectively, *P* = 0.61). Arterial PO_2_ was 175 ± 30 mmHg during Lactic Acid and 167 ± 34 during Glucose (*P* = 0.22).

Minute ventilation (8.7 ± 1.2 vs. 8.3 ± 1.2 L/minute, *P* <0.05) and alveolar ventilation (4 ± 0.3 vs. 3.8 ± 0.3 L/minute, *P* <0.05) were slightly (barely 5%) but significantly higher during Lactic Acid steps.

Body temperature was not different during Lactic Acid (38.0 ± 0.1°C) or Glucose (38.1 ± 0.1°C), *P* = 0.06. At all sample times all hemodynamic and respiratory parameters were comparable in both groups, except for a small increase in tidal volume which was required to compensate for the VCO_2_ rise (see Table 1)*.*

**Table 1:** Main hemodynamic and respiratory parameters

	**Glucose**	**Lactic acid**	***P***
Heart rate, bpm	91 ± 15	87 ± 18	*P* = 0.19
MAP, mmHg	74 ± 14	71 ± 12	*P* = 0.12
CVP, mmHg	10 ± 3	10 ± 4	*P* = 0.32
TV, ml	489 ± 52	513 ± 50	*P <0.05*
RR, breaths per minute	17.2 ± 1.2	17.2 ± 1.2	*P* = 0.32

bpm, beats per minute; CVP, Central Venous Pressure; MAP, Mean Arterial Pressure; RR, Respiratory Rate; TV, Tidal Volume. Mean ± standard deviation of data collected at first, second and fourth hour of each step.

## Discussion

In the present study, we evaluated the VCO_2_ changes in anesthetized pigs associated with lactic acid infusion. While maintaining a constant caloric intake, replacement of 50% of the caloric input with lactic acid, corresponding to an infusion of 2.5 m Eq/minute, caused an increase in total CO_2_ production of less than 5% (13 ml/minute).

This clinically small but statistically significant difference in total CO_2_ production may be due to several causes. First, a small increase in total VCO_2_ during lactic acid infusion is expected since the complete oxidation of lactate load produces 3.6% more CO_2_ than an amount of glucose of equal caloric content. Therefore, since in the present study lactic acid infusion provided 50% of the total caloric intake, a VCO_2_ increase of about 1.8% was expected. A variable proportion of the infused lactic acid may enter gluconeogenesis, and then possibly be stored as glycogen. The net result of this process will also cause a net increase in VCO_2_.

Another factor contributing to the VCO_2_ increase may result from the conversion of bicarbonate ions into CO_2_, caused by the increased systemic steady state levels of lactate and the resulting very mild metabolic acidosis. Indeed, although lactate levels remained lower than 3 mEq/l, a small reduction in arterial pH (of 0.023 pH units) was recorded. Such a systemic pH drop, promoting the «shift» of bicarbonate towards dissolved CO_2_, may have caused a transient increase in VCO_2_, despite a stable metabolic CO_2_ production. Failure to reach a steady state condition during lactic acid infusion might have contributed to the slight increase in VCO_2_. Overall, during lactic acid infusion, in order to maintain a constant arterial PCO_2_, a small increase in alveolar ventilation (5%) was required.

The infusion of lactic acid at the inlet of a membrane lung, by shifting the equilibrium towards the dissolved form of carbon dioxide, determines a significant increase of extracorporeal CO_2_ removal [12]. We have previously shown, in a similar animal model [14], that an infusion of 2.5 mEq/minute of lactic acid (the same rate as tested in the present study) at the inlet of a pediatric artificial lung (extracorporeal blood flow 0.25 L/minute), could increase extracorporeal CO_2_ removal by approximately 40 to 45 ml/minute (60 ± 9 vs. 101 ± 16 ml/minute, without and with lactic acid infusion, respectively), while the PaCO_2_ was maintained constant at 50 mmHg as in the current study. In that experiment we did not maintain a constant caloric intake, (caloric input increased from 43 to 90 Kcal/h when lactic acid was infused), hence, the total VCO_2_ was not evaluated.

In the current study, at variance, we kept the caloric input constant and we observed that lactic acid infusion slightly increases the total CO_2_ production (13 ml/minute with our experimental setting). We may, therefore, speculate that regional extracorporeal blood acidification at the inlet of the membrane lung may allow a substantial decrease in the patient’s ventilatory needs; actually, despite increasing the total CO_2_ production (13 ml/minute increase), it produces a significantly higher increase in extracorporeal CO_2_ removal (40 to 45 ml/minute increase). This assumption may be true only if, during lactic acid infusion, the total caloric intake is maintained constant.

The caloric input (around 2,330 kcal/day) applied in the present study, was based on literature and preliminary data [14,18]. The infusion of 2.5 mEq/minute of lactic acid (approximately 1,150 Kcal/day), representing around 50% of the daily energy requirement of our model, would provide a conspicuous fraction of the daily caloric needs of an ICU patient and, therefore, it should always be included in the energy balance.

The actual impact of lactic acid infusion, as part of an extracorporeal CO_2_ removal technique, on the nutritional balance is still to be determined. Lactate enters the glucose metabolism pathway after conversion to pyruvate; if a patient’s total caloric input is kept constant, we can assume that this pyruvate may follow the same metabolic pathway as the pyruvate produced from glucose metabolism. Furthermore, the insulin infusion rate required during the Glucose and Lactic acid steps to maintain a constant blood glucose concentration was similar and very low; hence, it is unlikely that insulin infusion may have altered the balance between glucose storage and glucose oxidation.

The choice of lactic acid to achieve blood acidification has several advantages: it is not toxic [17], it has a very fast metabolism and point-of-care testing of lactate is available in most ICUs. In our previous study we also demonstrated that a 48-hour lactic acid infusion, performed through an extracorporeal dialysis circuit, is safe and does not lead to any injury of red blood cells and major organs [14]. Blood acidification may be achieved by infusion of other metabolizable acids, such as citric or acetic acid; however, their anions are difficult to measure in the clinical setting. Moreover, their clearance is less predictable than that of lactate, at least for citric acid [19]. Furthermore, oxidation of equal caloric amounts of these acids produces more CO_2_ than lactic acid (see Table 2).

**Table 2:** **CO**_**2 **_**production from total oxidation of different substrates to produce 1 Kcal**

**Substrate**	**Kcal**	**Mmol of substrate**	**CO**_**2 **_**production (mmol)**	**CO**_**2 **_**production (ml)**
Glucose	1	1.48	8.9	199
Lactate	1	3.08	9.25	207
Acetate	1	4.93	9.85	220
Citrate	1	2.12	12.73	285

An alternative choice may be the utilization of non-metabolizable acids (for example, hydrochloric acid), which do not provide calories; however, their usage requires an extracorporeal removal of the infused anions which may significantly increase the complexity of the extracorporeal system [20,21].

Some limitations of the present study deserve to be discussed. First, our animal model may present some differences from humans: in a growing pig, even if anesthetized and paralyzed, the basal energy requirement and VCO_2_ per unit weight are far greater than those of an adult man [22]. Moreover, we cannot exclude the fact that different results would have derived from a critically ill animal model. Second, in our model, the arterial pH and bicarbonate ion concentrations are elevated, and may not closely represent the critical patients, even though the bicarbonate levels may be comparable to the one of chronic hypercapnic patients. Third, we did not investigate the metabolic pathway of infused lactate, which would have required the application of specific techniques (for example, use of isotopic carbon-labeled glucose or lactate) that are beyond the scope of the present investigation.

## Conclusions

Administration of 50% of the total caloric input with an infusion of lactic acid (2.5 mEq/minute) only marginally increased (less than 5%) the total CO_2_ production compared to an equal caloric load provided entirely by infusion of a 50% glucose solution. Since an equal rate of acidification, as shown in a previous study, increased the membrane lung removal by at least 50 ml/minute, we may speculate that blood acidification at the inlet of a membrane lung could be a promising technique to reduce the ventilatory needs.

## Key messages

• Infusion of lactic acid slightly increases the total CO_2_ production compared to an isocaloric infusion of glucose.

• An extracorporeal CO_2_ removal technique based on blood acidification with lactic acid infusion could be a promising technique to reduce the ventilatory needs, if the total caloric intake is kept constant.

## Abbreviations

ECCO2R: Extracorporeal CO_2_ removal; VA: Alveolar ventilation; VCO2: CO_2_ production; VD: Ventilation of the dead space; VE: Minute ventilation; VILI: Ventilation-Induced Lung Injury.

## Competing interests

Some procedures here described are part of a blood processing technique covered by patents or for which patents are pending: inventors: Antonio Pesenti, Nicolò Patroniti.

1). International patent application: PCT/EP 2008/003661; 7 May 2008

2). Italy: MI2007A000913; 7 May 2007

3). Italy: BO2012A000404; 26 July 2012; Bologna, Italy

## Authors’ contributions

AZ and MG participated in the design of the study, took part in the experiments, performed the statistical analysis and drafted the manuscript. PM, SR and VS participated in the design of the study and took part in the experiments. MC and MA took part in the experiments. VO took part in the experiments and data analysis. GB helped with the revision of the paper. NP and AP conceived of the study, and participated in its design and coordination, and helped to review the manuscript. All authors read and approved the final manuscript.
